# Vitamin B12 replacement strategies: a Brazilian delphi consensus

**DOI:** 10.1016/j.eclinm.2026.104205

**Published:** 2026-09-23

**Authors:** Ricardo V. Cohen, Randy Levinson, Otavio Baiocchi, João Eduardo N. Salles, Marcondes Cavalcante França, Tomas Navarro, Fabio Trujilho, José Maria Soares, Daniel Nakagawa, Marcelo Valente, Ricardo Barbuti, Raphael Ribeiro Spera, Fabio Moura, Cynthia Valerio, Carlos A. Schiavon, Fania Cristina dos Santos, Anna Carolina Batista Dantas, Andre Abdo, Marcelo Luis Steiner, Henri Augusto Korkes, Julianne Tannous Cordenonssi, Philip Bachour

**Affiliations:** a The Center for the Treatment of Obesity and Diabetes, Hospital Alemão Oswaldo Cruz, São Paulo, SP, Brazil; b Lai Wen Di, LLC, New York, NY, USA; c Hematology and Coagulation Group, Hospital Alemão Oswaldo Cruz, São Paulo, SP, Brazil; d Discipline of Endocrinology, Department of Internal Medicine, Faculty of Medical Sciences, Santa Casa de São Paulo, Brazilian Diabetes Society, São Paulo, SP, Brazil; e Department of Neurology, School of Medical Sciences, University of Campinas (UNICAMP), Campinas, SP, Brazil; f Department of Gastroenterology, Centro Especializado em Aparelho Digestivo, Hospital Alemão Oswaldo Cruz, University of São Paulo School of Medicine, São Paulo, SP, Brazil; g Brazilian Society of Endocrinology and Metabolism (SBEM), Centro de Diabetes e Endocrinologia da Bahia (CEDEBA), Salvador, BA, Brazil; h Discipline of Gynecology, Department of Obstetrics and Gynecology, Hospital das Clínicas, University of São Paulo School of Medicine, São Paulo, SP, Brazil; i Digestive Diseases Center, Hospital Alemão Oswaldo Cruz, Hospital das Clínicas, University of São Paulo School of Medicine, São Paulo, SP, Brazil; j Discipline of Geriatrics, Centro Universitário FMABC, Santo André, SP, Brazil; k Gastroenterology, Hospital das Clínicas, University of São Paulo School of Medicine, São Paulo, SP, Brazil; l Department of Neurology, Hospital das Clínicas, University of São Paulo School of Medicine, São Paulo, SP, Brazil; m Endocrinology and Metabolism, Hospital Universitário Oswaldo Cruz, University of Pernambuco, Instituto de Medicina Integral Prof. Fernando Figueira (IMIP), Recife, PE, Brazil; n Instituto Estadual de Diabetes e Endocrinologia do Rio de Janeiro (IEDE), Rio de Janeiro, RJ, Brazil; o BP - A Beneficência Portuguesa de São Paulo, Instituto Obesidade Brasil, São Paulo, SP, Brazil; p Discipline of Geriatrics and Gerontology, University of São Paulo, São Paulo, SP, Brazil; q Department of Surgery, Federal University of Rio Grande do Norte (UFRN), Natal, RN, Brazil; r Lymphoma and Hematology Center, Hospital Alemão Oswaldo Cruz, São Paulo, SP, Brazil; s Discipline of Gynecology, Centro Universitário FMABC, Santo André, SP, Brazil; t Obstetrics, Pontifícia Universidade Católica de São Paulo (PUC-SP), Sorocaba, SP, Brazil; u Myralis Indústria Farmacêutica Ltda, São Paulo, SP, Brazil; v Oncology and Hematology Center, Hospital Alemão Oswaldo Cruz, São Paulo, SP, Brazil

**Keywords:** Vitamin B12, Cobalamin, Delphi consensus, Oral supplementation, Sublingual, Intramuscular

## Abstract

**Background:**

Vitamin B12 deficiency is common in older adults, atrophic gastritis, and after bariatric surgery, and can cause irreversible neurological injury. Oral, sublingual, and intramuscular routes all correct serum concentrations, yet whether any route is superior for clinically meaningful outcomes is unclear, and international guidelines diverge. This consensus was convened to define where evidence is adequate, where it is lacking, and the priority research questions.

**Methods:**

This was a modified Delphi consensus study. The RAND/UCLA Appropriateness Method was applied through a two-stage process of 2 electronic rounds and an in-person meeting in São Paulo, Brazil. Panellists were recruited between 1 July 2025 and 15 July 2025. Thirty statements were voted on, presented as 31 graded items, using a binary (agree or disagree) format with a priori percentage-agreement grades (A+, 100%; A, 90%–99.9%; B, 80%–89.9%; C, 70%–79.9%; and D, 66%–69.9%). Draft statements, informed by a systematic review, were rated anonymously by a Brazilian multidisciplinary panel of 20 experts across haematology, gastroenterology, endocrinology, bariatric surgery, and geriatric medicine/neurology. Of 20 experts invited, all 20 agreed to participate and completed the electronic rounds (100%); 16 (80%) attended the in-person meeting. Patient representatives (n = 2) participated as non-voting observers. Grade A (at least 90% agreement) was the stopping rule. This consensus was not registered.

**Findings:**

In total, 30 statements across 6 modules were agreed, comprising 31 separately graded items as statement 1 was presented in two parts (1a and 1b); 22 of 31 items (71%) reached grade A+ (100% agreement) and 9 of 31 (29%) reached grade A (90%–99.9%). All routes were judged capable of normalising serum vitamin B12, but none showed proven superiority for symptom resolution, neurological recovery, functional status, or quality of life. Evidence gaps were greatest after bariatric surgery, in older adults with cognitive impairment or frailty, and in pernicious anaemia. Dosing, monitoring, and response definitions varied substantially.

**Interpretation:**

Vitamin B12 replacement across all routes remains guided primarily by biochemical rather than clinical evidence. Treatment should be individualised by aetiology, patient preference, feasibility, and access, with functional biomarkers and clinical assessment complementing serum levels. Adequately powered, long-term comparative trials using standardised, patient-centred outcomes are the priority.

**Funding:**

This work was funded by Myralis Indústria Farmacêutica Ltda, São Paulo, Brazil.

**Research in context**

**Evidence before this study**

Before convening this consensus, a companion systematic review was conducted to assemble the evidence comparing oral, sublingual, and intramuscular vitamin B12 replacement (PROSPERO CRD420251126832). PubMed, Embase, and Cochrane CENTRAL were searched from inception to July 18, 2025, without language or date restrictions; this search was updated on July 13, 2026 and yielded no additional eligible studies, combining vitamin B12 terms (cobalamin and its forms) with oral, sublingual, and intramuscular route terms. Eligible studies were comparative randomised trials and observational cohorts in adults with a replacement indication reporting serum vitamin B12 or clinical response; non-comparative studies, paediatric populations, reviews, and case reports were excluded. Risk of bias was assessed with RoB 2.0 and ROBINS-I. In total, 13 comparative studies were identified; the evidence was limited, heterogeneous, and at variable risk of bias, so a descriptive synthesis without pooling was performed. Most compared oral with intramuscular administration and reported broadly comparable short-term biochemical responses, with the sublingual route underrepresented. The most quantitative synthesis available, a 2024 network meta-analysis, ranked the intramuscular route highest for biochemical efficacy, with no significant difference between non-injectable routes. No study assessed symptom resolution, neurological recovery, functional status, or quality of life.

**Added value of this study**

To the best of our knowledge, this is the first structured, multidisciplinary consensus to grade vitamin B12 replacement against clinical rather than purely biochemical outcomes. Across the 30 consensus statements, the panel agreed that all routes normalise serum concentrations while none has demonstrated clinical superiority, and that this evidence gap is universal across routes rather than route-specific. It is most pronounced after bariatric surgery, in older adults with cognitive impairment or frailty, and in pernicious anaemia. The consensus also sets out a prioritised, outcome-focused research agenda.

**Implications of all the available evidence**

Current practice and guidelines extrapolate from biochemical normalisation to clinical recommendation without outcome-based trial data. Until adequately powered, long-term randomised trials with standardised, patient-centred endpoints are available, route selection should be individualised by aetiology, patient preference, feasibility, and access, with functional biomarkers and clinical assessment complementing serum concentrations alone. Priority should go to comparative effectiveness trials in the highest-need groups: patients after bariatric surgery, older adults with cognitive impairment or frailty, and those with pernicious anaemia. The immediate next steps are to validate these statements in other health systems and to design adequately powered comparative trials reporting symptom resolution, neurological recovery, functional status, and quality of life alongside biochemical response.

## Introduction

Vitamin B12 deficiency is common in several clinical populations, affecting approximately 6%–30% of older adults, 10%–30% of individuals with atrophic gastritis, and 5%–40% of patients after bariatric surgery.^1^, ^2^, ^3^ Clinical manifestations are heterogeneous and may be irreversible, including megaloblastic anaemia, peripheral neuropathy, subacute combined degeneration of the spinal cord, cognitive impairment, and dementia-like syndromes.^1^^,^^3^ Despite more than eight decades of clinical use and extensive publication, key questions regarding optimal vitamin B12 replacement remain unresolved. The existing evidence base is predominantly biochemical, focused on normalisation of serum vitamin B12 concentrations, with limited assessment of clinically meaningful outcomes such as symptom resolution, neurological recovery, functional status, or quality of life. Consequently, although multiple routes correct laboratory abnormalities, it remains unclear whether any approach confers superior long-term clinical benefit.^1^ This uncertainty is reflected in current clinical guidance. Major international guidelines, including those from the American Academy of Family Physicians (2017), the British Society for Haematology (2014), the National Institute for Health and Care Excellence (2024), and the Province of British Columbia (2023),^4^, ^5^, ^6^, ^7^ diverge in recommendations regarding route of administration, dosing, and monitoring. Such variability likely reflects limitations in the underlying evidence rather than substantive disagreement and is mirrored by wide variation in prescribing practices across clinicians and specialities.

A central limitation across all vitamin B12 replacement routes (oral, sublingual, and intramuscular) is the absence of robust evidence evaluating clinical outcomes. Sublingual therapy is supported by randomised controlled trials^8^^,^^9^ and nonrandomised comparative studies.^10^ Oral therapy relies on similarly limited randomised evidence, including the OB12 trial.^11^ Intramuscular administration, despite its historical role, also lacks randomised trials assessing clinical outcomes. In a 2024 network meta-analysis of 13 comparative studies (8 randomised trials; 4275 participants), intramuscular administration ranked highest for biochemical efficacy followed by sublingual and oral routes with no statistically significant differences between noninjectable approaches.^12^ Notably, none of these routes has been evaluated in randomised trials assessing symptom improvement, neurological recovery, or quality of life.

Current vitamin B12 replacement practices remain largely guided by achievement of biochemical targets rather than robust evidence of clinical benefit, despite the potential for irreversible neurological sequelae with delayed or inadequate treatment.^1^ This consensus was therefore convened to clarify where evidence is sufficient, where substantial uncertainty persists, and which questions require urgent, outcome-focused investigation to support more evidence-based use of oral, sublingual, and intramuscular vitamin B12.^1^

## Methods

### Study design and panel selection

This consensus was developed using the RAND/UCLA Appropriateness Method.^13^ Throughout, an indication for vitamin B12 replacement was defined as biochemically confirmed deficiency (serum vitamin B12 below the assay reference range), with or without supportive functional markers or clinical features, or a condition mandating replacement such as pernicious anaemia or post-bariatric status. The Delphi process was facilitated by an independent expert with formal training in consensus methodology and clinical expertise in endocrinology and metabolism. The consensus is reported in accordance with the Conducting and Reporting of Delphi Studies (CREDES) guidance.^14^

A multidisciplinary panel of 20 Brazilian experts was recruited based on their academic or leadership roles in professional societies, guideline panels, or clinical programmes related to haematology, gastroenterology, endocrinology, bariatric surgery, or geriatric medicine/neurology. Panellists were identified through national professional societies and academic networks and were invited individually by email between 1 July 2025 and 15 July 2025, to ensure representation across specialities and practice settings. The invitation described the purpose and scope of the consensus, the Delphi format, and the intention to publish the results. This was a modified Delphi consensus; no separate study protocol was registered or published, and the methodology is described in full in this section. Of 20 experts invited, all 20 agreed to participate (100%). All 20 completed the electronic Delphi rounds, and 16 of 20 (80%) attended the in-person consensus meeting. Vote totals per statement were 20 in the electronic rounds and 16 at the in-person meeting. All panellists were based in Brazil. The composition of the panel, including speciality and geographic location, is summarised in Table 1. In addition, two patient representatives with lived experience of vitamin B12 deficiency were recruited through a patient advocacy organisation and participated as observers. They contributed to the open panel deliberations by sharing the patient perspective on treatment burden and on outcomes that matter to patients, by helping to refine the wording of statements, and by informing the prioritisation of topics. They did not take part in the formal voting. Voting was restricted to the expert panel because the Delphi statements required appropriateness judgements on technical clinical and methodological questions for which structured ratings were sought from content experts; the patient representatives' input was incorporated qualitatively into the discussion and statement revision rather than through the numerical vote. This non-voting role is acknowledged as a limitation with respect to fully patient-led consensus development. In accordance with the Sex and Gender Equity in Research (SAGER) guidelines, the sex of panel members is reported in Table 1. Sex was not collected directly at panel formation; it was recorded retrospectively by the study team on the basis of panellists’ given names and publicly available professional records, and was verified by the corresponding author. Gender identity, age, years of professional experience, and race or ethnicity were not collected and are therefore not reported. Because the outputs of this consensus are expert-agreed statements rather than participant-level outcomes, the results could not be disaggregated or analysed by sex or gender, and the potential influence of panel sex composition on the statements cannot be quantified; this is noted as a limitation. The 20 panellists were drawn from multiple institutions and states of Brazil, supporting multidisciplinary and cross-regional representation, although the single-country composition limits sociodemographic and geographic diversity. No formal sample-size calculation applies to a Delphi consensus; panel size was guided by established methodological recommendations, which indicate that expert panels of approximately 10–20 participants are adequate to achieve stable consensus while maintaining disciplinary breadth and manageable group dynamics. A panel of 20 was chosen to ensure representation across the specialities relevant to vitamin B12 replacement, namely haematology, gastroenterology, endocrinology, bariatric and metabolic surgery, geriatric medicine, neurology, and gynaecology, and all 20 experts participated in both electronic rounds.

**Table 1:** Composition of the delphi panel.

#	Initials	Sex	Specialty/expertise	Institution	City/State	Country	In-person meeting
1	AA	M	Haematology	Hospital Alemão Oswaldo Cruz	São Paulo, SP	Brazil	Present
2	PB	M	Haematology/Oncology	Hospital Alemão Oswaldo Cruz	São Paulo, SP	Brazil	Present
3	OCGB	M	Haematology	Hospital Alemão Oswaldo Cruz	São Paulo, SP	Brazil	Remote
4	RCB	M	Gastroenterology	University of São Paulo	São Paulo, SP	Brazil	Present
5	ACBD	F	Bariatric & metabolic surgery	Federal University of Rio Grande do Norte (UFRN)	Natal, RN	Brazil	Present
6	RVC	M	Bariatric & metabolic surgery	Hospital Alemão Oswaldo Cruz	São Paulo, SP	Brazil	Present
7	FCS	F	Geriatric medicine	University of São Paulo	São Paulo, SP	Brazil	Present
8	MCFJ	M	Neurology	Department of Neurology, FCM-UNICAMP	Campinas, SP	Brazil	Present
9	HAK	M	Obstetrics (maternal–foetal)	PUC-SP	Sorocaba, SP	Brazil	Remote
10	FFM	M	Endocrinology	University of Pernambuco; IMIP	Recife, PE	Brazil	Present
11	DMN	M	Gastroenterology	Hospital Alemão Oswaldo Cruz; University of São Paulo	São Paulo, SP	Brazil	Present
12	TNR	M	Gastroenterology	University of São Paulo School of Medicine; Hospital Alemão Oswaldo Cruz	São Paulo, SP	Brazil	Remote
13	JENS	M	Endocrinology/Diabetes	Santa Casa de São Paulo School of Medical Sciences; Brazilian Diabetes Society	São Paulo, SP	Brazil	Remote
14	CAS	M	Bariatric & metabolic surgery	BP - A Beneficência Portuguesa de São Paulo; Instituto Obesidade Brasil	São Paulo, SP	Brazil	Present
15	JMSJ	M	Gynaecology	Hospital das Clínicas, FMUSP	São Paulo, SP	Brazil	Present
16	RRS	M	Neurology	University of São Paulo	São Paulo, SP	Brazil	Present
17	MLS	M	Gynaecology	Centro Universitário FMABC	Santo André, SP	Brazil	Present
18	FRT	M	Endocrinology (obesity/metabolism)	CEDEBA; Hospital da Obesidade, Salvador	Salvador, BA	Brazil	Present
19	MV	M	Geriatric medicine	Centro Universitário FMABC; Santa Casa de São Paulo	Santo André, SP	Brazil	Present
20	CMV	F	Endocrinology/Diabetes	IEDE - Instituto Estadual de Diabetes e Endocrinologia	Rio de Janeiro, RJ	Brazil	Present

Panellists are listed alphabetically by surname and identified by initials. Sex was recorded retrospectively by the study team from panellists' given names and publicly available professional records and verified by the corresponding author; it was not self-reported. Gender identity, age, years of professional experience, and race or ethnicity were not collected. All 20 panellists completed both electronic Delphi rounds; the in-person meeting column indicates those present in person (n = 16) and those participating remotely (n = 4). Two patient representatives attended as non-voting observers and are not listed.

SP = Sao Paulo; BA = Bahia; PE = Pernambuco; RJ = Rio de Janeiro; RN = Rio Grande do Norte.

All 20 panelists participated in the electronic Delphi rounds. Sixteen panelists attended the in-person meeting (80%). Four panelists participated remotely when feasible; therefore, vote totals varied by statement.

### Delphi process

Following preparation of the draft consensus document, an independent two-stage Delphi process was conducted. The first stage (“pre-meeting Delphi”) was performed electronically beginning on August 13, 2025. This panel used a binary (yes/no) voting format with percentage agreement thresholds, rather than the classical 1–9 appropriateness scale and disagreement index described in the original RAND/UCLA Appropriateness Method. This modification was chosen to simplify participation for a multidisciplinary, time-limited panel and to prioritise clear identification of statements with very high agreement. We acknowledge that, unlike the classical 1–9 appropriateness scale and its disagreement index, a binary format does not capture the degree of agreement, uncertainty, or dispersion among panellists, a trade-off we note in the Limitations.

Consensus grades were defined a priori based on percentage agreement: A+ (100%), A (90%–99.9%), B (80%–89.9%), C (70%–79.9%), and D (66%–69.9%). Statements with <66% agreement were considered to have failed consensus. Statements achieving A+ consensus were closed to further voting. Statements receiving an A grade or lower and for which feedback suggested the potential for improvement were revised by the Delphi facilitator and redistributed for a second electronic round. In total, two pre-meeting Delphi rounds were conducted.

The second stage (“in-person Delphi”) was held on October 17, 2025, in São Paulo, Brazil, with 16 panelists present. All remaining statements, including revised versions from the electronic rounds, were discussed sequentially. Statements not achieving A+ consensus were edited in real time based on panel discussion and re-voted anonymously using a smartphone-based voting system. If consensus thresholds were not met, further discussion and revision occurred until an acceptable grade was achieved. One additional statement was introduced and voted on during the in-person meeting. For transparency, both the percentage agreement and the total number of votes are reported for each statement. No further rounds were planned once all statements reached at least grade A (≥90% agreement), which served as the predefined stopping rule. The two-stage Delphi process is summarised in Fig. 1.

**Fig. 1:**
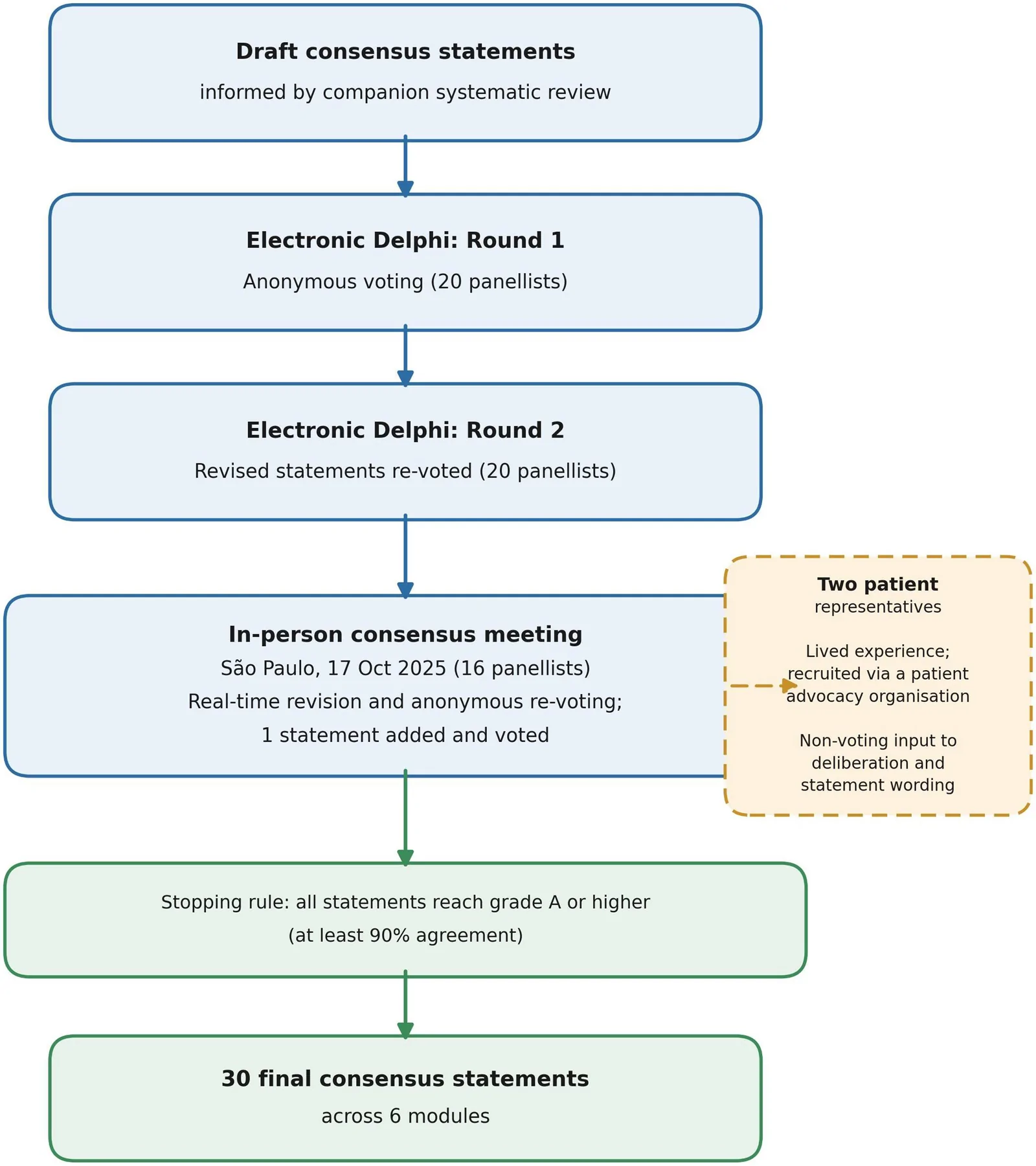
Study profile. Draft statements informed by the companion systematic review entered two electronic Delphi rounds followed by an in-person consensus meeting. Two patient representatives provided non-voting input that informed panel deliberation and statement revision; they did not participate in the formal voting. This figure 1 was generated in Python using Matplotlib version 3.10.8 solely for graphical presentation; Python was not used for data analysis or statistical testing. IM = intramuscular.

### Evidence synthesis and ranking metrics

Delphi statements were informed by a companion systematic review conducted specifically to provide the panel with a common, standardised evidence base before voting (Debone et al., submitted; PROSPERO CRD420251126832), which included randomised controlled trials, observational comparative studies, and recent network meta-analyses evaluating vitamin B12 supplementation routes. This search was updated on July 13, 2026. Before circulation to the panel, the draft statements were reviewed for clarity and unambiguous wording by the independent facilitator; no formal pilot testing with panellists was undertaken. For comparative ranking of biochemical efficacy across routes, results from network meta-analyses were summarised using SUCRA (surface under the cumulative ranking curve), a metric quantifying the probability that an intervention ranks among the most effective options for a given outcome on a scale from 0 to 100.^15^ Higher SUCRA values reflect ranking probability rather than effect size or clinical superiority. No meta-analysis was performed in this consensus; its outputs are agreement grades rather than pooled effect estimates, so quantitative measures used in meta-analysis or network meta-analysis, including funnel plots, Egger's test, I^2^, CINeMA, and network meta-analysis risk-of-bias tools, do not apply. The included comparative studies were not stratified by treatment phase and predominantly assessed initial biochemical correction, with sparse maintenance-phase comparative data. The specific studies and level of evidence underpinning each consensus statement are summarised in Supplementary Material S2.

### Ethics

This consensus did not involve patients, patient-level data, or any clinical intervention. Participants were healthcare professionals contributing expert opinion in their professional capacity, and no personal or health-related data were collected from panellists or from the two patient representatives who attended as non-voting observers. Formal research ethics committee approval was therefore not required. All panellists were invited individually, were informed of the purpose and scope of the consensus and of the intention to publish it, and participated voluntarily; agreement to take part constituted informed consent. The Declaration of Helsinki governs medical research involving human participants, their data, or their biological material, and therefore does not apply to this consensus; participation was nonetheless voluntary and informed, and the process is reported transparently.

### Statistical analysis

No inferential statistical testing was performed. Agreement was summarised descriptively as the percentage of panellists voting in favour of each statement, with the corresponding vote counts, and statements were assigned a priori agreement grades on that basis. Because the outputs are agreement grades rather than pooled effect estimates, no sensitivity, subgroup, or post-hoc analyses were undertaken, and measures used in meta-analysis or network meta-analysis do not apply. Voting was administered and tabulated on the electronic Delphi platform, and percentages and vote counts were verified against the underlying voting record in Microsoft Excel (Microsoft 365).

### Role of the funding source

This consensus was funded by Myralis Indústria Farmacêutica Ltda, São Paulo, Brazil, which financed the electronic Delphi platform and paid the honoraria and travel costs of the panel and the facilitator. Several safeguards protected the independence of evidence appraisal, statement development, and voting. The evidence base was an independently registered systematic review (PROSPERO CRD420251126832) conducted to a pre-specified protocol, rather than material supplied by the funder. Panellists were identified through national professional societies and academic networks, with no funder involvement in their selection. Consensus grades, thresholds, and the stopping rule were defined a priori, and all voting was anonymous. The funder had no role in the design or conduct of the Delphi, the drafting, wording of, or voting on statements, or the analysis and interpretation of results, and had no access to the voting data. One author (JTC) is an employee of the funder and contributed only to review and editing of the manuscript, taking no part in statement development or voting. The facilitator received consulting fees and travel support from the funder; his role was methodological and non-voting, so independence was protected by the process described above rather than by his financial independence from the funder. The statements contain no brand-specific or product-specific recommendations, and the conclusions emphasise the limitations of the evidence and the absence of proven superiority for any route. The academic corresponding author accessed and verified the voting data and had sole responsibility for the decision to submit.

## Results

The updated search, performed on July 13, 2026, yielded no additional studies that were eligible for inclusion. Of 20 experts invited, 20 of 20 agreed to participate (100%); all 20 completed the two electronic rounds and 16 of 20 (80%) attended the in-person meeting, with 4 of 20 (20%) participating remotely. The process produced 30 numbered consensus statements across six thematic modules, presented as 31 graded items because statement 1 comprises parts 1a and 1b; part 1b was introduced during the in-person meeting and voted on at that meeting.

Consensus was reached for every item. In total, 22 of 31 items (71%) reached grade A+ (100% agreement) and 9 of 31 (29%) reached grade A (90%–99.9%); no item was graded B or lower. Twenty-four statements were finalised in the electronic rounds, 23 at the first round and 1 at the second, and 7 at the in-person meeting (items 1a, 1b, 7, 11, 19, 20, and 25).

The median number of voting rounds per statement was 1 (range 1–3), and 4 statements were re-voted in the second electronic round (statements 1, 7, 19, and 21). Per-statement agreement, vote counts at each stage, and dissenting votes are provided in Supplementary Material S1. The full set of consensus statements, with agreement grades and vote counts, is presented in Table 2.

**Table 2:** Consensus statements, agreement grades, and voting.

Statement	Grade	Agreement (%)	Rounds	Votes (n)
Module 1: Routes of administration and efficacy				
1a. All three routes (oral, sublingual, IM) can effectively normalise serum B12 in most adults.	A+	100	2	16
1b. Higher oral dose may be required in atrophic gastritis or malabsorption.	A+	100	1	16
3. IM achieves biochemical correction; clinical benefit data limited.	A	95	1	20
4. Insufficient evidence to determine long-term clinical superiority of any route.	A	95	1	20
6. In patients without malabsorption, oral appears comparable to IM.	A	95	1	20
7. For pernicious anaemia, IM remains preferred; large oral doses can be efficacious.	A+	100	3	16
19. Route choice should be individualized based on aetiology and patient preference.	A+	100	3	16
Module 2: Biomarkers and therapeutic endpoints				
2. Evidence primarily assessed biochemical response; limited clinical outcome assessment.	A+	100	1	20
5. Serum B12 alone has uncertain clinical significance; functional biomarkers needed.	A	95	1	20
8. RCTs show comparable biochemical responses; high heterogeneity in dosing/outcomes.	A+	100	1	20
9. IM regimens vary dramatically in frequency, dose, duration across studies.	A+	100	1	20
24. No consensus on monitoring frequency for B12, MMA, or homocysteine.	A+	100	1	20
25. Inconsistent response/normalization definitions limit comparability.	A+	100	2	16
26. Maintenance regimens highly variable; not well-supported by trial data.	A+	100	1	20
Module 3: Sublingual supplementation				
11. Sublingual shows similar short-term biochemical efficacy in available RCTs (low certainty).	A+	100	2	16
12. Insufficient evidence for sublingual on clinical symptoms/quality of life.	A+	100	1	20
13. Sublingual administration may be acceptable for some patients, although comparative evidence regarding acceptance, access, and patient-centred outcomes is limited.	A	95	1	20
14. No direct comparison of long-term neurological outcomes: sublingual vs. IM.	A+	100	1	20
15. No clinical or biochemical advantage of sublingual over oral demonstrated.	A+	100	1	20
16. Studies comparing oral/sublingual used varied formulations/doses; limits generalizability.	A	95	1	20
17. No consensus on optimal sublingual dosing; no pharmacokinetic data.	A+	100	1	20
18. Comparative adherence/tolerability between oral/sublingual underexplored.	A	95	1	20
Module 4: Special populations				
20. Data on sublingual after bariatric surgery sparse; limited to small observational study.	A+	100	2	16
21. Oral B12 may be effective post-bariatric; higher doses may be required.	A	95	2	20
22. Lack of evidence for B12 supplementation in elderly with cognitive decline/frailty.	A+	100	1	20
Module 5: Dosing and monitoring protocols				
23. Optimal dosing strategies not standardized; vary substantially across studies.	A+	100	1	20
Module 6: Safety, adherence, and patient preferences				
10. Patient preference and adherence may vary by route; available comparative evidence remains limited.	A	95	1	20
27. Adverse events rarely reported; likely underrecognized.	A+	100	1	20
28. Sublingual appears well tolerated; systematic safety assessment lacking.	A+	100	1	20
29. Some patients may prefer non-injectable routes, but robust evidence regarding patient preferences is insufficient.	A+	100	1	20
30. Discomfort and access requirements associated with intramuscular administration could potentially influence adherence; however, comparative adherence data remain limited.	A+	100	1	20

Thirty numbered statements are presented as 31 graded items, since statement 1 comprises parts 1a and 1b. Agreement grades were defined a priori: A+, 100%; A, 90%–99.9%; B, 80%–89.9%; C, 70%–79.9%; and D, 66%–69.9%. Rounds indicates the number of voting rounds required; Votes indicates the number of panellists voting on that item (20 in the electronic rounds, 16 at the in-person meeting).

IM = intramuscular; SL = sublingual; QoL = quality of life; MMA = methylmalonic acid.

## Discussion

This Delphi consensus confirms that all three vitamin B12 replacement routes (oral, sublingual, and intramuscular) can reliably normalise serum vitamin B12 levels, while highlighting the persistent absence of evidence that any route is superior for clinically meaningful outcomes such as symptom resolution, neurological recovery, functional improvement, or quality of life. As a result, clinicians can achieve biochemical targets using any route but remain unable to determine which strategy optimises patient-centred outcomes over the long term.

This limitation applies uniformly across routes. Oral therapy shows biochemical equivalence to intramuscular in patients without malabsorption, while sublingual demonstrates short-term efficacy in limited studies, but, as the panel unanimously recognised, none shows clinical superiority (Statements 3, 4, 6, 11, 12, and 15). Comparative analyses based on biochemical rankings, including recent network meta-analysis, do not address this gap and should not be interpreted as evidence of clinical benefit (Statements 2, 4). Serum vitamin B12 rises with pharmacological dosing regardless of clinical benefit and is therefore a limited marker of treatment effect, useful mainly for confirming adherence on oral therapy; the magnitude of the rise also differs by cobalamin form, being greater with cyanocobalamin than with hydroxocobalamin, without implying clinical superiority. The high agreement recorded here therefore reflects shared recognition of these evidence gaps and cautious, uncertainty-acknowledging statements, and should not be read as strength of the underlying evidence.

The consensus further highlights that vitamin B12 supplementation guidelines and clinical practices frequently extrapolate biochemical findings to clinical recommendations in the absence of robust outcome-based trial data. This disconnect between biochemical success and clinical evidence reflects a broader pattern across the field.

Evidence gaps are particularly pronounced in vulnerable populations. Reflecting these universal limitations across routes, the panel identified particular deficits in older adults with cognitive impairment or frailty (Statement 22). Post-patients after bariatric surgery remain understudied across all routes, with limited evidence supporting oral therapy and sparse data for sublingual formulations (Statements 20, 21). Despite lifelong dependence on supplementation, patients with pernicious anaemia are underrepresented in modern outcome-focused trials (Statement 7).

Substantial heterogeneity in dosing strategies, maintenance regimens, and monitoring practices further compounds uncertainty. No consensus exists regarding optimal dosing or monitoring frequency for serum vitamin B12, methylmalonic acid, or homocysteine (Statements 9, 17, 23, 24, and 26). Definitions of treatment response vary widely, limiting comparability across studies (Statement 25). Safety, tolerability, adherence, and patient preference data are also limited, with adverse events likely underreported and comparative adherence studies lacking across all modalities (Statements 10, 27–30).

The panel was convened in a single country (Brazil) and recruited through professional networks, potentially limiting generalisability. The recommendations may reflect Brazilian practice patterns, product availability, and reimbursement, and may not transfer directly to other health systems; international validation would strengthen external validity. Patient representatives contributed to discussions but lacked voting rights, so statements addressing patient preference, adherence, and acceptability reflect clinician judgement informed qualitatively by patient input rather than direct patient-reported data. The modified RAND/UCLA approach, including the use of percentage-based agreement thresholds rather than the classical 1–9 scale, may limit comparability with other Delphi studies and is described in detail in the Methods Section; a binary vote also does not quantify the degree of agreement or any residual disagreement beyond the reported percentages.

This consensus documents persistent evidence gaps in vitamin B12 supplementation. Over decades, research has prioritised biochemical normalisation while largely neglecting clinical outcomes that matter to patients. This gap is universal rather than route-specific and limits the ability of clinicians and guideline developers to make evidence-based decisions regarding route selection, dosing, monitoring, and long-term management.

This consensus contributes 31 graded statements synthesising the available evidence and expert judgement across all vitamin B12 replacement routes, a systematic documentation of universal evidence gaps extending beyond biochemical normalisation, and a prioritised research agenda focused on patient-centred outcomes in high-risk populations.

Accordingly, the consensus supports individualised treatment decisions based on aetiology, patient preference, feasibility, and access (Statement 19), while emphasising transparent communication regarding current evidence limitations. Serum vitamin B12 concentrations alone should not be interpreted as definitive indicators of clinical benefit, and functional biomarkers and clinical assessment should inform management when appropriate (Statement 5). Future progress will require well-designed randomised trials comparing all routes using standardised, patient-centred clinical outcomes and sufficient follow-up to capture neurological and functional effects.

This Delphi consensus provides a rigorous synthesis of current knowledge and uncertainty surrounding vitamin B12 supplementation. Across 30 statements, the panel achieved a high level of agreement, reflecting not resolution of long-standing clinical questions, but broad recognition of persistent and clinically relevant evidence gaps.

A central conclusion is that oral, sublingual, and intramuscular vitamin B12 replacement can all achieve biochemical correction, yet none has demonstrated superiority in clinically meaningful outcomes. Consequently, current practice across all routes continues to rely primarily on biochemical normalisation rather than outcome-based evidence.

The consensus further identifies substantial heterogeneity in dosing, maintenance regimens, monitoring strategies, and therapeutic endpoints, along with limited data on safety, adherence, and patient preferences. These limitations apply across all routes and are particularly pronounced in vulnerable populations, including older adults with cognitive impairment, patients after bariatric surgery, and individuals with pernicious anaemia.

Ultimately, the most important contribution of this consensus is the identification of a systemic research gap in vitamin B12 supplementation. Until high-quality randomised trials with standardised, patient-centred clinical outcomes are available, treatment decisions should be individualised and guided by aetiology, patient preference, feasibility, and access, with functional biomarkers and clinical assessment informing management rather than reliance on serum vitamin B12 concentrations alone. To support dissemination and implementation in national practice, these statements will be submitted to the major relevant Brazilian professional societies with a view to endorsement.

## Contributors

RVC conceived the consensus, provided overall supervision, and served as one of the 20 voting panellists. RSL, an independent expert with formal training in consensus methodology, facilitated the Delphi process, designed the rounds, drafted, and revised the statements between rounds, and curated the voting data. The expert panellists (OCGB, JENS, MCFJ, TNR, FRT, JMSJ, DMN, MV, RCB, RRS, FFM, CMV, CAS, FCS, ACBD, AA, MLS, HAK, and PB) rated the statements across rounds and contributed to in-person deliberation and revision. JTC reviewed and edited the manuscript. RVC and RSL accessed and verified the voting data. All authors reviewed the manuscript critically, approved the final statements and the version submitted, and RVC had final responsibility for the decision to submit.

## Data sharing statement

The round-by-round voting record, including the per-statement percentage agreement, vote counts at each stage, and the revisions made between rounds, is provided in Supplementary Material S1. Further materials are available from the corresponding author on reasonable request. No bespoke software or source code was developed for this consensus; voting was administered on a commercial electronic Delphi platform and the results were tabulated and verified using standard spreadsheet software, so no code is available to share. Individual panellists’ votes are not shared because voting was conducted anonymously and panellists were not asked to consent to release of individually identifiable votes.

## Declaration of interests

This consensus was funded by Myralis Indústria Farmacêutica Ltda (Myralis), São Paulo, Brazil, the manufacturer of a vitamin B12 product. JTC is an employee of Myralis (Senior Manager, Strategy and Innovation), the funder of this work. Several authors disclose relationships with Myralis arising from the consensus process, as detailed below.

RSL received consulting fees and travel support from Myralis for facilitating the Delphi process. JENS received support for the present work from Myralis and has served on a Myralis advisory board; he also reports consulting fees from Myralis, Novo Nordisk, Eli Lilly, Merck, EMS, Abbott ADC, Eurofarma, and Boehringer Ingelheim, honoraria from Novo Nordisk, Eli Lilly, Merck, EMS, Abbott ADC, Eurofarma, and Boehringer Ingelheim, and unpaid leadership roles with the Brazilian Diabetes Society (SBD), ABESO, and FLASO. FRT received support for the present work, consulting fees, travel support, and advisory-board participation from Myralis, and reports unpaid roles with Asociacao Brasileira de Estudos Sobre a Obesidade, Federacao Latino Americana Sobre a Obesidade, and Sociedade Brasileira de Endocrinologia e Metabolismo. OCGB received a consultancy honorarium from Myralis for the present work, and reports consulting fees and honoraria from Roche, Abbvie, and AstraZeneca and advisory-board participation for Takeda, MSD, and Abbvie. CAS received support for the present work and honoraria from Myralis. ACBD received support for the present work from Myralis (honorarium and travel for the consensus meeting). RRS received support for the present work from Myralis and reports honoraria from Eli Lilly and Aché. MCFJ, DMN, and MV received support for the present work from Myralis. JMSJ received grant funding from Myralis. HAK received honoraria from Myralis. TNR reports honoraria from Aché, Apsen, Myralis, and Takeda. FCS reports honoraria from Myralis, Amgen, Adium, and GSK and unpaid committee roles with SBGG, SBED, and ABRASSO.

MLS reports medical-writing support for the present manuscript and consulting fees from Eurofarma, EMS, Abbott, and Pfizer and honoraria from Astellas, Bayer, Libbs, Merck, and FMQ. CMV reports consulting fees from Eli Lilly, Novo Nordisk, Chiesi, Merck, and EMS, honoraria from Eli Lilly, Novo Nordisk, Chiesi, Merck, Abbvie, EMS, Boehringer Ingelheim, and Eurofarma, travel support from AstraZeneca, Eli Lilly, Novo Nordisk, Chiesi, Merck, and EMS, advisory-board participation for Eli Lilly and Novo Nordisk, and a board role with ABESO. FFM reports consulting fees from Novo Nordisk, honoraria from AstraZeneca, Boehringer Ingelheim, Eli Lilly, EMS, Merck, and Novo Nordisk, travel support from AstraZeneca and Novo Nordisk, advisory-board participation for Novo Nordisk, and an unpaid directorship of the Brazilian Society of Endocrinology and Metabolism. RCB reports grants from Takeda, Eurofarma, Apsen, Mantecorp, and Aché, consulting fees from Aché, Mantecorp, Apsen, Takeda, and Eurofarma, honoraria from Aché, Mantecorp, Apsen, Sanofi, Takeda, EMS, and Eurofarma, payment for expert testimony from Aché and Apsen, travel support from Aché, Apsen, and Sanofi, and advisory-board participation for Aché and Apsen. RVC reports research grants to his institution from Johnson & Johnson Medical Brazil and Medtronic Brazil, honoraria from Johnson & Johnson Medical Brazil, Medtronic, Novo Nordisk, Eli Lilly, and Currax, and participation on scientific advisory boards for Morphic Medical, Johnson & Johnson, and Novo Nordisk. PB and AA declare no competing interests.

## Appendix A Supplementary data

Supplementary Material

Translation Abstract

## References

[1] Stabler S.P. (2013). Clinical practice. Vitamin B12 deficiency. N Engl J Med.

[2] Ha J., Kwon Y., Kwon J.W. (2021). Micronutrient status in bariatric surgery patients receiving postoperative supplementation per guidelines: insights from a systematic review and meta-analysis of longitudinal studies. Obes Rev.

[3] Karhunen P., Tuomisto S., Goebeler S., Martiskainen M., Kok E. (2025). Common occurrence of atrophic gastritis in an ageing non-hospitalised population: an autopsy study. Age Ageing.

[4] Langan R.C., Goodbred A.J. (2017). Vitamin B12 deficiency: recognition and management. Am Fam Physician.

[5] Devalia V., Hamilton M.S., Molloy A.M., British Committee for Standards in Haematology (2014). Guidelines for the diagnosis and treatment of cobalamin and folate disorders. Br J Haematol.

[6] (2024). Vitamin B12 Deficiency in Over 16s: Diagnosis and Management.

[7] https://www2.gov.bc.ca/gov/content/health/practitioner-professional-resources/bc-guidelines/vitamin-b12.

[8] Sharabi A., Cohen E., Sulkes J., Garty M. (2003). Replacement therapy for vitamin B12 deficiency: comparison between the sublingual and oral route. Br J Clin Pharmacol.

[9] Parry-Strong A., Langdana F., Haeusler S., Weatherall M., Krebs J. (2016). Sublingual vitamin B12 compared with intramuscular injection in patients with type 2 diabetes treated with metformin: a randomized trial. N Z Med J.

[10] Bensky M.J., Ayalon-Dangur I., Ayalon-Dangur R. (2019). Comparison of sublingual vs. intramuscular administration of vitamin B12 for the treatment of patients with vitamin B12 deficiency. Drug Deliv Transl Res.

[11] Sanz-Cuesta T., Escortell-Mayor E., Cura-Gonzalez I. (2020). Oral versus intramuscular administration of vitamin B12 for vitamin B12 deficiency in primary care: a pragmatic randomized non-inferiority trial (OB12). BMJ Open.

[12] Abdelwahab O.A., Abdelaziz A., Diab S. (2024). Efficacy of different routes of vitamin B12 supplementation: a systematic review and network meta-analysis. Ir J Med Sci.

[13] Khodyakov D., Grant S., Korger J., Bauman M. (2023).

[14] Jünger S., Payne S.A., Brine J., Radbruch L., Brearley S.G. (2017). Guidance on Conducting and REporting DElphi Studies (CREDES) in palliative care: recommendations based on a methodological systematic review. Palliat Med.

[15] Salanti G., Ades A.E., Ioannidis J.P.A. (2011). Graphical methods and numerical summaries for presenting results from multiple-treatment meta-analysis. J Clin Epidemiol.

