# Supplementary material for: Vitamin B12 replacement strategies: a Brazilian delphi consensus
Source: eClinicalMedicine. 2026 Sep 23;100:104205. doi: 10.1016/j.eclinm.2026.104205 (PMC13634271; doi:10.1016/j.eclinm.2026.104205)
Supplement: Supplementary file 1 [file mmc1.pdf]

Supplementary Material

Vitamin B12 Replacement Strategies: A Brazilian Delphi Consensus

Manuscript eclinm-D-26-03219, eClinicalMedicine

Contents

Supplementary Material S1. Round-by-round Delphi voting record 2

Supplementary Material S2. Consensus statements mapped to supporting evidence and level of evidence 7

Page numbers refer to this combined supplementary file.

Supplementary Material S1. Round-by-round Delphi voting record

Vitamin B12 Replacement Strategies: A Brazilian Delphi Consensus (eclinm-D-26-03219). Per-statement agreement at each stage. Two pre-meeting electronic rounds were completed by all 20 panellists (n=20); the in-person meeting on 17 October 2025 was attended by 16 of the 20 panellists (n=16), who re-voted anonymously. Agreement grades were defined a priori: A+ = 100%, A = 90 to 99.9%, B = 80 to 89.9%, C = 70 to 79.9%, D = 66 to 69.9%; the stopping rule was grade A (at least 90%). Statement wordings shown here are those voted on by the panel.

#	Consensus statement (as voted)	Round 1 (electronic, n=20)	Round 2 (electronic, n=20)	In-person (n=16)	Final grade (%)	Rounds
**1a**	All three administration routes (oral, sublingual, intramuscular) can be effective in normalizing serum vitamin B12 levels in most adults with deficiency.	90% (18/20); 2 disagreed	75% (15/20); 5 disagreed	100% (16/16)	A+ (100%)	2
**1b**	A higher oral dose of B12 may be required to treat a subset of patients with deficiency, including those with atrophic gastritis or any other form of malabsorption.	- (added in-person)	-	100% (16/16)	A+ (100%)	1
**2**	Evidence to date comparing the administration routes of vitamin B12 primarily evaluated the biochemical response, with limited assessment of clinical outcomes, such as symptom resolution or neurological recovery.	100% (20/20)	-	-	A+ (100%)	1
**3**	Intramuscular vitamin B12 achieves biochemical correction in a wide range of deficiency etiologies, though data supporting clinical benefit are limited.	95% (19/20); 1 disagreed	-	-	A (95%)	1
**4**	There is insufficient high-quality evidence to determine the long-term clinical superiority of any particular route of administration of vitamin B12.	95% (19/20); 1 disagreed	-	-	A (95%)	1
**5**	The use of serum vitamin B12 concentration alone as a therapeutic endpoint is of uncertain clinical significance, as functional biomarkers of vitamin B12	95% (19/20); 1 disagreed	-	-	A (95%)	1

	deficiency (e.g., methylmalonic acid (MMA), homocysteine) have been inconsistently reported.					
**6**	In patients without malabsorptive conditions, oral vitamin B12 supplementation appears comparable to intramuscular administration in achieving biochemical correction.	95% (19/20); 1 disagreed	-	-	A (95%)	1
**7**	Among patients with pernicious anemia, intramuscular vitamin B12 remains the preferred route due to concerns regarding intrinsic factor–dependent absorption, despite limited robust, long-term data from comparisons to other means of delivery, including that a large enough oral dose can be sufficiently absorbed via passive diffusion to be efficacious.	80% (16/20); 4 disagreed	75% (15/20); 5 disagreed	100% (16/16)	A+ (100%)	3
**8**	Randomized controlled trials show comparable short-term biochemical responses between oral and intramuscular supplementation, but with high heterogeneity in dosing and outcome definitions.	100% (20/20)	-	-	A+ (100%)	1
**9**	Intramuscular regimens are more variable in frequency, dose, and duration across studies, complicating cross-study comparisons.	100% (20/20)	-	-	A+ (100%)	1
**10**	Patient preference, adherence, and access to care may favor oral therapy in real-world settings, but comparative adherence data are scarce.	95% (19/20); 1 disagreed	-	-	A (95%)	1
**11**	Sublingual supplementation demonstrates similar short-term biochemical efficacy	95% (19/20); 1 disagreed	-	100% (16/16)	A+ (100%)	2

	to intramuscular routes in a single RCT, though with low certainty due to risk of bias and imprecision.					
**12**	There is insufficient evidence to assess whether sublingual vitamin B12 improves clinical symptoms or quality of life compared to intramuscular administration.	100% (20/20)	-	-	A+ (100%)	1
**13**	Sublingual routes may offer advantages in terms of patient acceptance and healthcare access, but these outcomes have not been rigorously evaluated.	95% (19/20); 1 disagreed	-	-	A (95%)	1
**14**	No studies to date have directly compared long-term neurological or functional outcomes between sublingual and intramuscular vitamin B12 supplementation therapy.	100% (20/20)	-	-	A+ (100%)	1
**15**	Current evidence does not demonstrate any clinical or biochemical advantage of sublingual over oral vitamin B12 supplementation.	100% (20/20)	-	-	A+ (100%)	1
**16**	Studies to date comparing oral and sublingual vitamin B12 used varied formulations, doses, and administration frequencies, limiting generalizability.	95% (19/20); 1 disagreed	-	-	A (95%)	1
**17**	There is no consensus on optimal dosing for sublingual supplementation, and no studies to date have included pharmacokinetic data.	100% (20/20)	-	-	A+ (100%)	1
**18**	Comparative adherence and tolerability between oral and sublingual formulations remain underexplored.	95% (19/20); 1 disagreed	-	-	A (95%)	1
**19**	The choice of route of administration of vitamin B12 supplementation should be individualized	90% (18/20); 2 disagreed	90% (18/20); 2 disagreed	100% (16/16)	A+ (100%)	3

	based on the etiology of deficiency, including surgical, autoimmune, and drug-induced causes.					
**20**	Data supporting sublingual vitamin B12 use after metabolic and bariatric surgery are sparse and primarily limited to a small observational study.	100% (20/20)	-	100% (16/16)	A+ (100%)	2
**21**	Although the data is limited, oral supplementation with vitamin B12 may be effective in patients with food-cobalamin malabsorption and after metabolic and bariatric surgery, though higher doses may be required.	90% (18/20); 2 disagreed	95% (19/20); 1 disagreed	-	A (95%)	2
**22**	There is a lack of evidence guiding vitamin B12 supplementation strategies in elderly patients with cognitive decline or frailty.	100% (20/20)	-	-	A+ (100%)	1
**23**	Optimal dosing strategies for all routes—particularly sublingual—are not standardized and vary across studies.	100% (20/20)	-	-	A+ (100%)	1
**24**	There is no consensus on the frequency of monitoring serum vitamin B12, MMA, or homocysteine after supplementation.	100% (20/20)	-	-	A+ (100%)	1
**25**	Inconsistent definitions of ‘response’ and biochemical ‘normalization’ across studies limit comparability and hinder clinical interpretation.	95% (19/20); 1 disagreed	-	100% (16/16)	A+ (100%)	2
**26**	Maintenance regimens for intramuscular and oral vitamin B12 supplementation remain highly variable and are not well supported by trial data.	95% (19/20); 1 disagreed	-	-	A+ (100%)	1
**27**	Adverse events related to vitamin B12 supplementation are rarely	100% (20/20)	-	-	A+ (100%)	1

	reported and are likely underrecognized.					
**28**	Sublingual vitamin B12 supplementation appears well tolerated, though few studies have systematically assessed tolerability or side effects.	100% (20/20)	-	-	A+ (100%)	1
**29**	Patient preferences may favor non-injectable routes, especially in chronic deficiency, but structured preference studies are lacking.	100% (20/20)	-	-	A+ (100%)	1
**30**	Intramuscular therapy may be associated with lower adherence due to injection-related discomfort and access barriers to continued healthcare.	100% (20/20)	-	-	A+ (100%)	1

Notes. Statement 1 was split into 1a and 1b; 1b was introduced and voted at the in-person meeting. Statements re-voted in Round 2 (electronic): 1, 7, 19, 21. Statements finalised at the in-person meeting (n=16): 1a, 1b, 7, 11, 19, 20, 25. Vote counts and dissent are shown as agreement% (agree/total).

Statements 10, 13, 29, and 30 were subsequently reworded in response to peer review; the wording shown in this table is the wording voted on by the panel, and the final revised wording appears in the main text and in Supplementary Material S2. Per-statement free-text comments, where provided, are held in the individual panellist questionnaires and can be appended on request.

Supplementary Material S2. Consensus statements mapped to supporting evidence and level of evidence

Vitamin B12 Replacement Strategies: A Brazilian Delphi Consensus (eclinm-D-26-03219). Evidence is drawn from the companion systematic review (Debone et al.; PROSPERO CRD420251126832). Level of evidence follows the OCEBM 2011 Levels of Evidence: Level 2, randomised trial(s); Level 3, non-randomised controlled cohort; Level 4, case series or poor-quality cohort; Level 5, mechanism-based or expert reasoning. The 30 consensus statements are presented as 31 graded items, since statement 1 comprises parts 1a and 1b. Statements citing specific trials or cohorts are mapped to the corresponding studies; statements that assert an absence of direct comparative evidence are identified as resting on expert consensus (Level 5), consistent with the review's central finding.

#	Consensus statement	Supporting evidence	Study design	OCEBM level
**1a**	All three routes (oral, sublingual, IM) can effectively normalize serum B12 in most adults.	Oral vs IM RCTs (Kuzminski 1998, Bolaman 2003, Castelli 2011, Sanz-Cuesta 2020); SL vs oral RCTs (Sharabi 2003, Sidrar 2024); SL vs IM (Parry-Strong 2016 RCT, Bensky 2019 obs.)	RCTs + observational	2
**1b**	Higher oral dose may be required in atrophic gastritis or malabsorption.	No direct comparative trial in this subgroup; extrapolated from absorption physiology	Expert consensus	5
**2**	Evidence primarily assessed biochemical response; limited clinical outcome assessment.	Characterisation of the evidence base (companion systematic review)	Expert consensus / narrative	5
**3**	IM achieves biochemical correction; clinical benefit data limited.	IM arms of oral vs IM RCTs (Kuzminski 1998, Bolaman 2003, Castelli 2011, Sanz-Cuesta 2020)	RCTs (biochemical)	2
**4**	Insufficient evidence to determine long-term clinical superiority of any route.	No study assessed long-term clinical superiority	Expert consensus (absence of evidence)	5
**5**	Serum B12 alone has uncertain clinical significance; functional biomarkers needed.	Methodological observation across included studies	Expert consensus	5
**6**	In patients without malabsorption, oral appears comparable to IM.	Oral vs IM RCTs (Kuzminski 1998, Bolaman 2003, Castelli 2011, Sanz-Cuesta 2020)	RCTs	2
**7**	For pernicious anemia, IM remains preferred; large oral doses can be efficacious.	No included study specifically enrolled pernicious anaemia; reflects clinical convention, with indirect support from oral vs IM biochemical equivalence	Expert consensus (indirect)	5
**8**	RCTs show comparable biochemical responses; high heterogeneity in dosing/outcomes.	Oral vs IM RCTs (as above)	RCTs	2
**9**	IM regimens vary dramatically in frequency, dose, duration across studies.	Methodological observation across included studies	Expert consensus	5
**10**	Patient preference and adherence may vary by route; available comparative evidence remains limited.	Preference data in one trial (Metaxas 2017); no comparative adherence data	RCT (partial) + expert consensus	4

**11**	Sublingual shows similar short-term biochemical efficacy in available RCTs (low certainty).	Parry-Strong 2016 (sublingual vs IM RCT; low certainty, risk of bias and imprecision)	Single RCT	3
**12**	Insufficient evidence for sublingual on clinical symptoms/quality of life.	No study assessed clinical symptoms or QoL for this comparison	Expert consensus (absence of evidence)	5
**13**	Sublingual administration may be acceptable for some patients, although comparative evidence regarding acceptance, access, and patient-centred outcomes is limited.	No direct comparative evidence on acceptance/access outcomes	Expert consensus	5
**14**	No direct comparison of long-term neurological outcomes: sublingual vs. IM.	No study identified	Expert consensus (absence of evidence)	5
**15**	No clinical or biochemical advantage of sublingual over oral demonstrated.	SL vs oral RCTs (Sharabi 2003, Sidrar 2024)	RCTs	2
**16**	Studies comparing oral/sublingual used varied formulations/doses; limits generalizability.	Methodological observation (Sharabi 2003, Sidrar 2024)	Expert consensus	5
**17**	No consensus on optimal sublingual dosing; no pharmacokinetic data.	No study identified	Expert consensus (absence of evidence)	5
**18**	Comparative adherence/tolerability between oral/sublingual underexplored.	No direct comparative evidence	Expert consensus	5
**19**	Route choice should be individualized based on etiology and patient preference.	Clinical reasoning; no comparative trial of individualised strategies	Expert consensus	5
**20**	Data on sublingual after bariatric surgery sparse; limited to small observational study.	Shakeri-Leidenmuhler 2013 (post-gastric-bypass, sublingual vs IM; small, non-randomised)	Observational	4
**21**	Oral B12 may be effective post-bariatric; higher doses may be required.	Post-surgery oral vs IM cohorts (Ramos 2021, Teja 2025); Adachi 2000 (post-gastrectomy)	Observational	3
**22**	Lack of evidence for B12 supplementation in elderly with cognitive decline/frailty.	No study identified in this population	Expert consensus (absence of evidence)	5
**23**	Optimal dosing strategies not standardized; vary substantially across studies.	Methodological observation across included studies	Expert consensus	5
**24**	No consensus on monitoring frequency for B12, MMA, or homocysteine.	No study addressed monitoring frequency	Expert consensus (absence of evidence)	5
**25**	Inconsistent response/normalization definitions limit comparability.	Methodological observation across included studies	Expert consensus	5
**26**	Maintenance regimens highly variable; not well-supported by trial data.	No trial evaluated maintenance regimens directly	Expert consensus (absence of evidence)	5
**27**	Adverse events rarely reported; likely underrecognized.	Reporting-gap observation across included studies	Expert consensus	5

**28**	Sublingual appears well tolerated; systematic safety assessment lacking.	Limited tolerability reporting (SL studies)	Observational / expert consensus	4
**29**	Some patients may prefer non-injectable routes, but robust evidence regarding patient preferences is insufficient.	No structured preference study	Expert consensus (absence of evidence)	5
**30**	Discomfort and access requirements associated with intramuscular administration could potentially influence adherence; however, comparative adherence data remain limited.	No direct comparative adherence evidence	Expert consensus	5

Companion review studies: Kuzminski 1998, Adachi 2000, Bolaman 2003, Sharabi 2003, Castelli 2011, Shakeri-Leidenmuhler 2013, Parry-Strong 2016, Metaxas 2017, Sanz-Cuesta 2020, Bensky 2019, Ramos 2021, Sidrar 2024, Teja 2025.
