# Supplementary material for: Vitamin B12 replacement strategies: a Brazilian delphi consensus
Source: eClinicalMedicine. 2026 Sep 23;100:104205. doi: 10.1016/j.eclinm.2026.104205 (PMC13634271; doi:10.1016/j.eclinm.2026.104205)
Supplement: Supplementary file 2 [file mmc2.docx]

**The following translations in Portuguese were submitted by the authors and we reproduce them as supplied. They have not been peer reviewed. Our editorial processes have only been applied to the original abstract in English, which should serve as reference for this manuscript.**

**Resumo traduzido (português do Brasil)**

**Estratégias de Reposição de Vitamina B12: Um Consenso Delphi Brasileiro**

**Contexto**

A deficiência de vitamina B12 é frequente em idosos, na gastrite atrófica e após cirurgia bariátrica, e pode causar lesão neurológica irreversível. As vias oral, sublingual e intramuscular corrigem as concentrações séricas, mas não está claro se alguma via é superior para desfechos clinicamente relevantes, e as diretrizes internacionais divergem. Este consenso foi convocado para definir onde a evidência é adequada, onde ela é insuficiente e quais são as questões de pesquisa prioritárias.

**Métodos**

Trata-se de um estudo de consenso Delphi modificado. O Método de Adequação RAND/UCLA foi aplicado em um processo de duas etapas, com 2 rodadas eletrônicas e uma reunião presencial em São Paulo, Brasil. Os painelistas foram recrutados entre 1 e 15 de julho de 2025. Trinta afirmativas foram votadas, apresentadas como 31 itens graduados, em formato binário (concorda ou discorda), com graus de concordância percentual definidos a priori (A+, 100%; A, 90% a 99,9%; B, 80% a 89,9%; C, 70% a 79,9%; D, 66% a 69,9%). As afirmativas preliminares, fundamentadas em uma revisão sistemática, foram avaliadas de forma anônima por um painel multidisciplinar brasileiro de 20 especialistas em hematologia, gastroenterologia, endocrinologia, cirurgia bariátrica e geriatria/neurologia. Dos 20 especialistas convidados, todos os 20 aceitaram participar e completaram as rodadas eletrônicas (100%); 16 (80%) participaram da reunião presencial. Dois representantes de pacientes participaram como observadores sem direito a voto. O grau A (concordância de pelo menos 90%) foi o critério de encerramento. Este consenso não foi registrado.

**Resultados**

No total, foram acordadas 30 afirmativas em 6 módulos, correspondendo a 31 itens graduados separadamente, uma vez que a afirmativa 1 foi apresentada em duas partes (1a e 1b); 22 dos 31 itens (71%) atingiram o grau A+ (concordância de 100%) e 9 dos 31 (29%) atingiram o grau A (90% a 99,9%). Todas as vias foram consideradas capazes de normalizar a vitamina B12 sérica, mas nenhuma demonstrou superioridade comprovada para resolução de sintomas, recuperação neurológica, estado funcional ou qualidade de vida. As lacunas de evidência foram maiores após cirurgia bariátrica, em idosos com comprometimento cognitivo ou fragilidade e na anemia perniciosa. As definições de posologia, monitoramento e resposta variaram substancialmente.

**Interpretação**

A reposição de vitamina B12, em todas as vias, permanece orientada principalmente por evidência bioquímica e não clínica. O tratamento deve ser individualizado conforme a etiologia, a preferência do paciente, a viabilidade e o acesso, com biomarcadores funcionais e avaliação clínica complementando os níveis séricos. Ensaios comparativos de longo prazo, com poder estatístico adequado e desfechos padronizados e centrados no paciente, são a prioridade.

**Financiamento**

Este trabalho foi financiado pela Myralis Indústria Farmacêutica Ltda, São Paulo, Brasil.

**Palavras-chave**

vitamina B12; cobalamina; consenso Delphi; suplementação oral; sublingual; intramuscular
